# Temporal trends and weight-class differences in takedown density in professional mixed martial arts: a retrospective analysis of 8,461 UFC bouts, 1997–2025

**DOI:** 10.1186/s13102-026-01804-8

**Published:** 2026-06-18

**Authors:** Kui Ma, Kang Li, Zhe Zhu, Hui Tian Shao

**Affiliations:** 1 Universiti Putra Malaysia (UPM), Selangor Darul Ehsan, Serdang, 43400 Malaysia; 2 Department of Lifestyle Sports, Dongshin University, Naju-si, Jeollanam-do Republic of Korea

**Keywords:** Mixed martial arts, UFC, Takedown density, Grappling performance, Performance analysis, Weight class, Quantile benchmarks

## Abstract

**Background:**

Takedowns are a central grappling action in mixed martial arts (MMA), but long-term evidence on time-normalized takedown output across competitive eras and weight classes remains limited. This study examined takedown density (TDpM; successful takedowns per minute) as a descriptive performance indicator in professional MMA.

**Methods:**

We analyzed publicly available UFC bout-level records from 1997 to 2025, comprising 8,461 bouts and 16,922 fighter–bout observations. TDpM was calculated as successful takedowns divided by bout duration in minutes. Temporal trends were examined separately for men and women, with sensitivity analyses excluding bouts shorter than 90 s and excluding pre-2002 men’s observations. Weight-class differences were evaluated using Kruskal–Wallis tests with Dunn–Holm post hoc comparisons. Division-specific percentile values were calculated as descriptive TDpM benchmarks, and winner–loser differences in TDpM, takedown success rate (TD%), and control time per successful takedown (CTRL/TD) were examined exploratorily.

**Results:**

Men’s annual mean TDpM declined significantly across 1997–2025 (β = −0.00426 takedowns·min⁻¹·year⁻¹, *r* = − 0.753, *p* < 0.001), and the negative direction remained in the 2002–2025 sensitivity analysis (β = −0.00202, *r* = − 0.712, *p* < 0.001). Women’s TDpM showed no significant trend across 2013–2025 (β = −0.00252, *r* = − 0.394, *p* = 0.183). Men’s TDpM differed significantly across weight classes (Kruskal–Wallis *p* < 0.001), although effect sizes were small (ε² ≈ 0.005–0.007); women’s between-division differences were not significant. Winners showed higher TDpM (median 0.067 vs. 0.000 takedowns·min⁻¹), TD% (50.0% vs. 20.0%), and CTRL/TD (99.75 vs. 78.88 s) than losers.

**Conclusions:**

TDpM provides a simple, time-normalized descriptive indicator of takedown-related performance in professional MMA. Men’s TDpM declined over time and differed modestly across weight classes, whereas women’s patterns were comparatively stable in the available sample. Percentile benchmarks and winner–loser contrasts offer descriptive reference values, but TDpM should not be interpreted as a causal determinant of victory or a training-prescription threshold. Future studies incorporating video-coded context and training data are needed to clarify its broader applied value.

**Supplementary Information:**

The online version contains supplementary material available at https://doi.org/10.1186/s13102-026-01804-8.

## Background

Performance in mixed martial arts (MMA) is multidimensional and depends on the integration of striking, clinching, grappling, and defensive actions [1, 2]. The broader concept of external load is well established in sport science and athlete monitoring [3–6]. Combat-sport studies have examined workload using accelerometry, time-motion analysis, lactate responses, and inertial-sensor approaches [7–9]. Quantifying total external load in MMA, however, requires integrating information across striking, clinching, grappling, defensive actions, locomotor activity, and other physical work, which is beyond the scope of bout-level public competition data [1, 3, 5, 8]. The present study therefore does not aim to quantify total external load. Instead, we focus on one operationally defined and time-normalized component of competition output: takedown density.

Among grappling-related actions, takedowns and the subsequent control sequence constitute a major technical-tactical pathway that shapes bout tempo and influences competitive outcomes [10–13]. Previous research has characterized MMA performance using technical-tactical indicators, time-motion variables, phase-specific actions, and outcome-related performance profiles [10–14]. However, evidence on time-normalized takedown density across weight classes and over long competitive eras remains limited. Time-normalization is particularly relevant given that bout durations vary substantially across MMA contests, which limits the comparability of raw counts across bouts of different lengths [7, 14]. In addition, technical preferences and bout pacing patterns differ across sex, round, and weight division, and UFC tactical patterns may evolve across competitive eras, making division-stratified and era-sensitive analysis important for descriptive benchmarking [15–19].

The aim of this retrospective analysis was to characterize takedown density (TDpM) as a descriptive, time-normalized performance indicator in professional MMA using a large historical UFC dataset. Within this descriptive scope, the study did not seek to establish a training-prescription model or test causal mechanisms of victory. We addressed three specific research questions: (i) what are the temporal trends in TDpM across men (1997–2025) and women (2013–2025); (ii) how does TDpM differ across sex- and division-stratified weight classes; and (iii) as an exploratory outcome-related analysis, do bout winners and losers differ in TDpM, takedown success rate (TD%), and control time per successful takedown (CTRL/TD)?

## Methods

### Data source and sample

This retrospective study used the publicly available Kaggle dataset “UFC 2025 Dataset: Fights, Fighters & Events” [20]. The dataset is released under a CC0 Public Domain license and is an openly released compilation of UFC competition records covering events from 1994 to 2025 and was originally compiled from publicly accessible UFC Stats records. It contains multiple CSV files including event-level metadata, fighter-level information, and bout-level technical statistics.

For the present study, we used bout-level and fighter-bout-level variables required to calculate takedown density and related grappling indicators, including bout date, sex, weight class, bout duration, winner-loser status, successful takedowns, attempted takedowns, and control time. Technical variables were used as recorded in the source dataset. We did not independently re-code video footage; therefore, all takedown-related variables should be interpreted as source-recorded competition statistics rather than independently verified video-coded events.

The analytical window covered 1997–2025 for men and 2013–2025 for women, corresponding to the available UFC competition history represented in the cleaned dataset for each sex. Records were included if they had valid bout duration, non-missing takedown statistics, identifiable sex, and a recognized UFC weight class. After data cleaning, 8,461 bouts were retained and converted into 16,922 fighter-bout observations, including 15,146 men’s observations and 1,776 women’s observations. Catchweight observations were retained in overall analyses but were not presented as a separate fixed division in the descriptive benchmark tables because catchweight does not correspond to a stable weight class.

The dataset contains publicly available professional-sport records and aggregate competition statistics. No private, medical, paywalled, or non-public personal data were used. The Kaggle source dataset is released under a CC0 Public Domain license, permitting unrestricted reuse.

### Data extraction and quality control

Data extraction, cleaning, and statistical analysis were conducted using Python 3.x. The source CSV files were imported, harmonized, and converted from bout-level records into fighter-bout observations where appropriate. The cleaning workflow included checking bout duration, sex, weight class, takedown success counts, takedown attempt counts, control time, and winner-loser status. Records with missing or invalid bout duration or missing takedown statistics were excluded from the analytical sample.

To assess data-processing accuracy from the source CSV files into the analytical dataset, a random 5% subset of retained bouts (*n* = 423) was re-checked using an independent extraction script and compared against the primary analytical dataset. The compared fields included bout duration, weight class, winner-loser status, successful takedowns, attempted takedowns, and control time. No discrepancies were identified across the checked fields. This procedure verified internal data-processing reproducibility from the same source CSV files; it does not constitute an independent video-coding reliability assessment of the underlying UFC Stats records.

Because this study relied on a secondary dataset compiled from publicly available UFC Stats records, the technical variables were not independently re-coded from original fight videos. This means that the validity of successful takedown counts, attempted takedowns, and control time depends on the source-recorded statistics. This reliance on source-recorded competition statistics is acknowledged as a methodological limitation.

### Metric definition and computation

Takedown density (TDpM; takedowns per minute) was defined as the number of successful takedowns divided by bout duration in minutes:

$$\mathrm{TDpM}=\frac{\mathrm{TDsucc}}{\mathrm{Tbout}}$$

where TDsucc denotes the number of successful takedowns recorded in the source dataset and Tbout denotes bout duration in minutes.

In this study, TDpM is interpreted as a time-normalized takedown performance indicator and a grappling-related output-density measure [14, 21]. It should not be interpreted as a comprehensive measure of total external load in MMA, because total external load would also include striking, clinching, defensive actions, locomotor activity, and other technical-tactical components [1, 3–5, 8].

Takedown success rate (TD%) was calculated as:

$$\mathrm{TD}\%=(\frac{\mathrm{TDsucc}}{\mathrm{TDatt}})\times 100$$

where TDatt denotes the number of attempted takedowns. TD% was calculated only for fighter-bout observations with at least one takedown attempt. TD% values are reported as percentages. Control time per successful takedown (CTRL/TD) was calculated as total control time divided by the number of successful takedowns and was calculated only for observations with at least one successful takedown. Control time was converted into seconds before calculating CTRL/TD.

To reduce inflation of density-based estimates from extremely short bouts, a sensitivity analysis excluding bouts shorter than 90 s was performed. This specification was used to evaluate whether the main TDpM patterns were robust to the exclusion of very short contests that could produce artificially high per-minute values.

### Statistical analysis

Distributional characteristics of TDpM were examined using histograms, Q-Q plots, and the Shapiro-Wilk test. TDpM was zero-inflated and right-skewed across most divisions; non-parametric procedures were therefore used as the primary inferential approach. Analyses were conducted separately for men and women because the UFC women’s divisions were introduced later and had a shorter observation window. Descriptive statistics were used to summarize sample characteristics and TDpM distributions. Because TDpM was zero-inflated and right-skewed, distributional summaries emphasized medians, interquartile ranges, and percentile-based thresholds rather than means alone.

Annual mean TDpM was calculated to describe temporal trends. Linear regression was then used to estimate the annual change in mean TDpM, with calendar year as the predictor and annual mean TDpM as the outcome. The regression slope, correlation coefficient, and p value were reported for each temporal specification. For men, temporal trends were examined for the full historical window from 1997 to 2025. To address potential rule-era comparability concerns, an additional sensitivity analysis excluding pre-2002 observations was performed to exclude the early pre-unified-rules period and the transition toward the modern unified-rules era. For women, temporal trends were examined from 2013 to 2025, corresponding to the introduction and development of women’s divisions in the UFC dataset.

Weight-class differences in TDpM were evaluated using the Kruskal-Wallis test because TDpM distributions were non-normal, zero-inflated, and heterogeneous across divisions [22]. Epsilon-squared was reported as an effect-size estimate for Kruskal-Wallis tests [23]. When the omnibus test was significant, Dunn’s post hoc pairwise comparisons with Holm correction were used to identify pairwise differences while controlling the family-wise error rate [24, 25]. To reduce instability in distributional estimates from very small groups, inferential weight-class comparisons were restricted to divisions with at least 30 fighter-bout observations. This threshold was used to limit small-sample instability rather than to imply formal measurement reliability assessment.

Winner-loser differences in grappling-related indicators were examined for TDpM, TD%, and CTRL/TD. Because these variables were skewed, group differences were summarized using medians and interquartile ranges. These comparisons were interpreted as exploratory outcome-related comparisons rather than causal evidence that takedown density determines bout outcome.

Sex- and division-specific P50, P75, and P90 values were calculated as descriptive quantile benchmarks for TDpM. These quantiles were used to characterize the distribution of takedown density within each sex and weight class. They should be interpreted as descriptive performance-analysis references rather than prescriptive training thresholds [14, 21].

All analyses were performed using Python 3.x with pandas, NumPy, SciPy, statsmodels, scikit-posthocs, and matplotlib. Statistical significance was set at *p* < 0.05.

## Results

### Sample overview

A total of 8,461 UFC bouts were included and converted into 16,922 fighter-bout observations, comprising 15,146 men’s observations and 1,776 women’s observations. The temporal analysis covered 1997–2025 for men and 2013–2025 for women. Sensitivity analyses excluding bouts shorter than 90 s were conducted to evaluate whether TDpM patterns were influenced by very short contests. For men, an additional rule-era sensitivity analysis excluding pre-2002 observations was conducted to evaluate whether the full-period pattern was influenced by the early pre-unified-rules period.

**Fig. 1:**
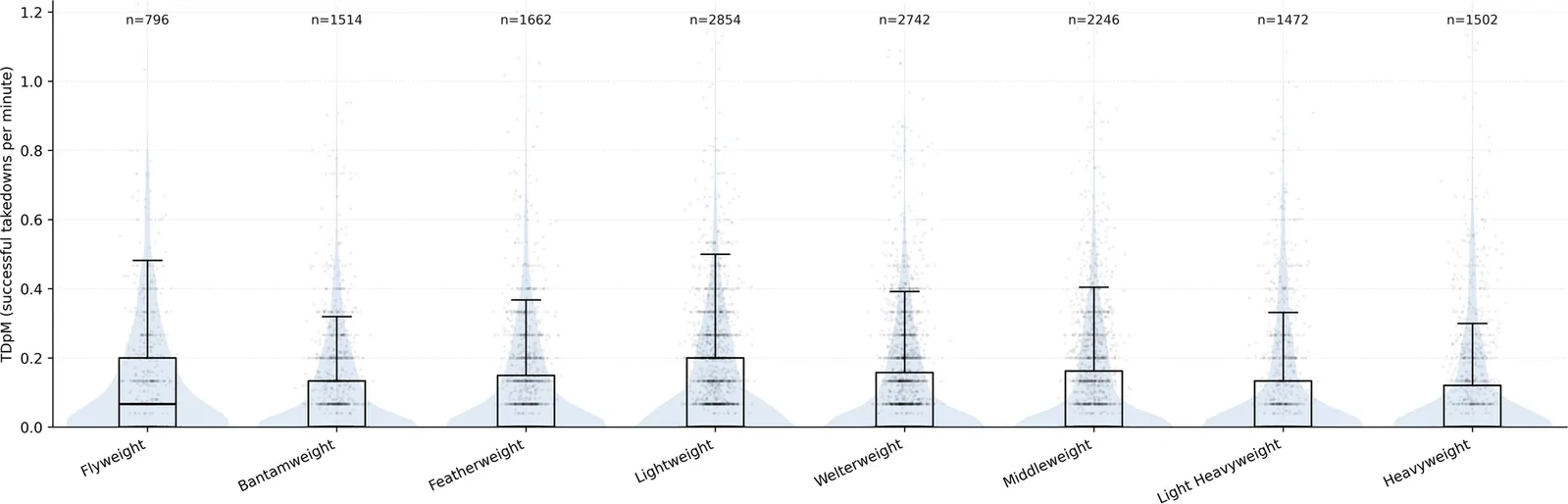
Distribution of takedown density across men’s weight classes. Raincloud plots show TDpM distributions across men’s UFC weight classes. Points represent individual fighter–bout observations, boxes indicate medians and interquartile ranges, and shaded density areas show distributional shape. Sample sizes are shown above each division

**Fig. 2:**
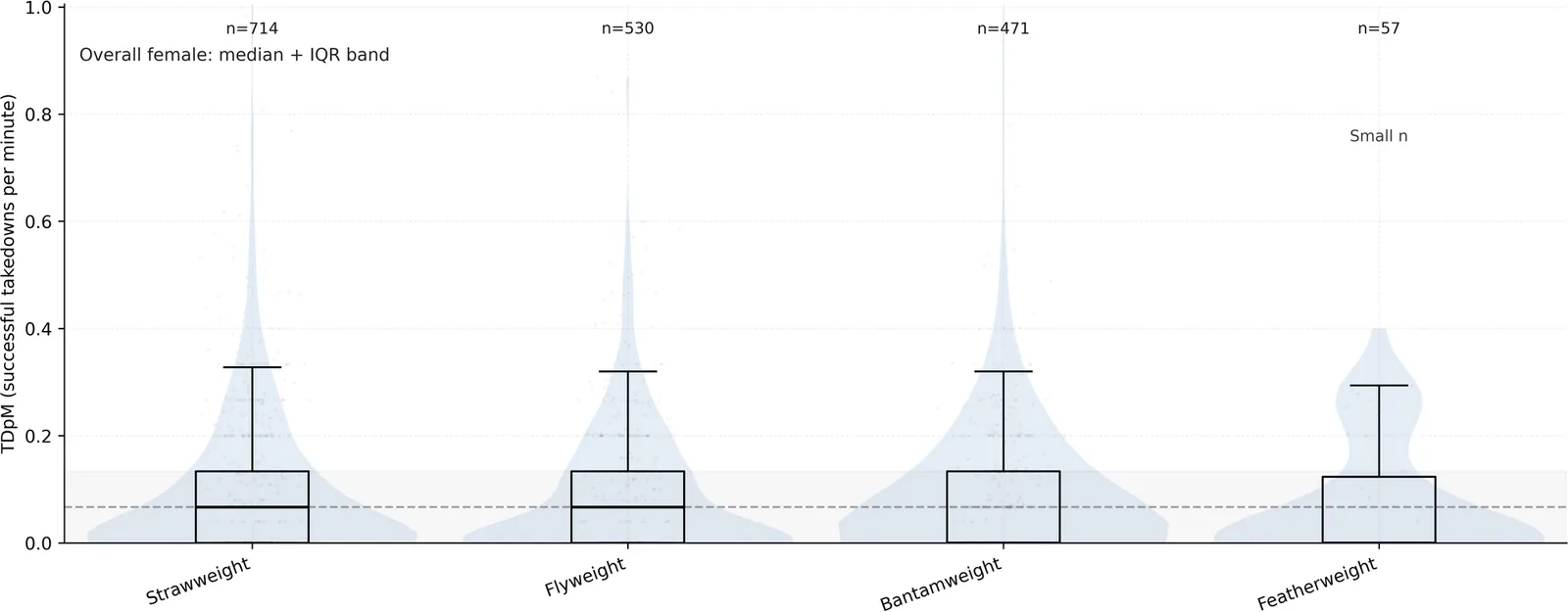
Distribution of takedown density across women’s weight classes. Raincloud plots show TDpM distributions across available women’s UFC weight classes. Points represent individual fighter–bout observations, boxes indicate medians and interquartile ranges, and shaded density areas show distributional shape. Sample sizes are shown above each division. The dashed horizontal line and shaded band summarize the overall female median and interquartile range

TDpM distributions were zero-inflated and right-skewed, with a substantial proportion of fighter-bout observations recording no successful takedown. Accordingly, medians, interquartile ranges, and percentile-based summaries were used alongside inferential tests. Men’s and women’s TDpM distributions across weight classes are shown in Figs. 1 and 2, respectively.

### Temporal trends in TDpM

**Fig. 3:**
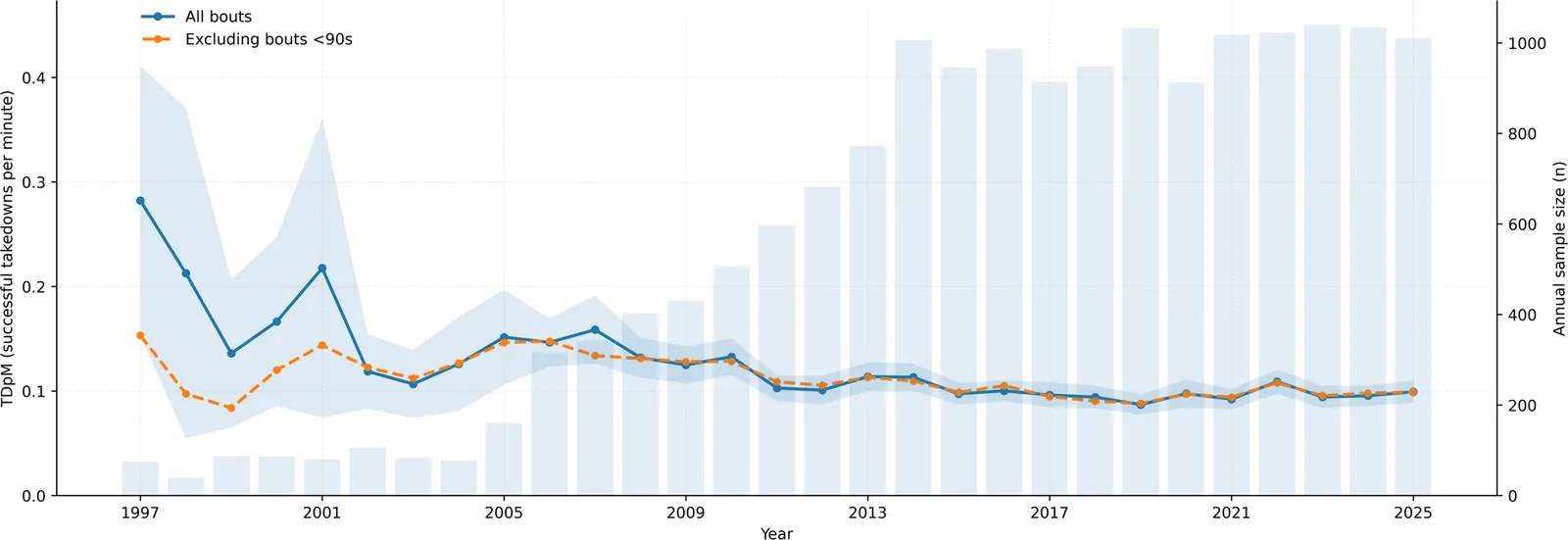
Annual trends in men’s takedown density, 1997–2025. Lines show annual mean TDpM for all bouts and for the sensitivity specification excluding bouts shorter than 90 s. The shaded band indicates variability around the annual trend, and background bars show annual sample size

Annual mean TDpM was examined separately for men and women. For men, TDpM showed a declining annual trend across the 1997–2025 observation window (Fig. 3; Table 1). The direction of the trend remained negative after excluding bouts shorter than 90 s. In the rule-era sensitivity analysis, the 2002–2025 specification also showed a negative and statistically significant annual trend in both the full sample and the specification excluding bouts shorter than 90 s.

**Table 1:** Temporal trend regression results for TDpM (Annual Mean TDpM as the Outcome)

Sex	Year range	Specification	Years (*n*)	Slope β	*r*	*p*
Men	1997–2025	Full sample	29	−0.00426	−0.753	< 0.001
Men	1997–2025	Excluding bouts < 90 s	29	−0.00187	−0.750	< 0.001
Men	2002–2025	Full sample	24	−0.00202	−0.712	< 0.001
Men	2002–2025	Excluding bouts < 90 s	24	−0.00189	−0.783	< 0.001
Women	2013–2025	Full sample	13	−0.00252	−0.394	0.183
Women	2013–2025	Excluding bouts < 90 s	13	−0.00122	−0.305	0.311

β represents the annual rate of change in mean TDpM (takedowns per minute). Results are computed at the annual mean level and should be interpreted as descriptive temporal trends rather than correlations computed at the individual bout level. The 2002–2025 specifications are rule-era sensitivity analyses excluding pre-2002 observations

**Fig. 4:**
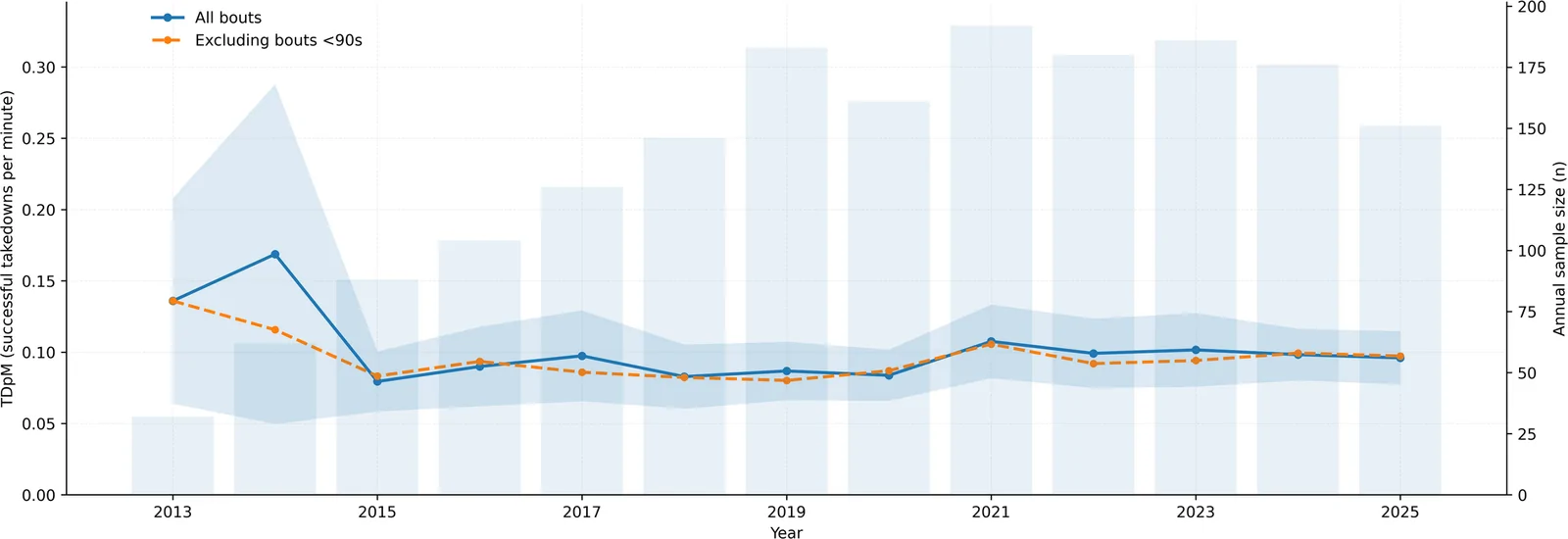
Annual trends in women’s takedown density, 2013–2025. Lines show annual mean TDpM for all bouts and for the sensitivity specification excluding bouts shorter than 90 s. The shaded band indicates variability around the annual trend, and background bars show annual sample size

For women, annual mean TDpM was examined from 2013 to 2025 (Fig. 4; Table 1). The estimated annual slope was negative but not statistically significant in either the full-sample specification or the specification excluding bouts shorter than 90 s. Given the shorter observation window and smaller annual sample size, the women’s temporal results should be interpreted descriptively.

### Weight-class differences in TDpM

Weight-class differences in TDpM were evaluated using the Kruskal-Wallis test, with epsilon-squared reported as the effect-size estimate (Table 2). In men, TDpM differed significantly across weight classes in both the full-sample specification and the specification excluding bouts shorter than 90 s. The overall effect sizes were small, indicating that between-division differences explained only a limited proportion of total TDpM variability.

**Table 2:** Weight-class differences in TDpM (Kruskal-Wallis test, exact p value, and ε² effect size; full sample and sensitivity analysis excluding bouts < 90 s; *n* ≥ 30)

Sex	Specification	Min *n*	k	*N*	H	*p*	ε²
Men	Full sample	30	9	14,940	105.773	2.81e-19	0.00655
Men	Excl. <90 s	30	9	13,802	71.296	2.71e-12	0.00459
Women	Full sample	30	4	1776	3.391	0.335	0.00022
Women	Excl. <90 s	30	4	1718	2.260	0.520	< 0.00001

k denotes the number of divisions included in the Kruskal-Wallis test. ε² denotes the effect size of the Kruskal-Wallis test

Post hoc Dunn-Holm comparisons indicated that significant pairwise differences were mainly driven by lower TDpM values in the heavier divisions relative to several lighter and mid-weight divisions. In particular, Heavyweight and Light Heavyweight tended to show lower TDpM than Bantamweight, Featherweight, Flyweight, Lightweight, Welterweight, and Middleweight in the primary sensitivity specification. Most adjacent weight-class comparisons were not statistically significant.

In women, TDpM did not differ significantly across weight classes in either the full-sample specification or the specification excluding bouts shorter than 90 s. Effect sizes were close to zero, indicating minimal between-division explanatory value in the available women’s sample. These results are reported descriptively given the smaller sample size and fewer available women’s divisions.

Dunn’s post hoc pairwise comparisons with Holm correction are reported in Supplementary Material S1. The primary pairwise specification excluded bouts shorter than 90 s and included divisions with at least 30 fighter-bout observations.

### Descriptive TDpM quantile benchmarks

Sex- and division-specific TDpM percentiles were calculated to summarize the distribution of takedown density across weight classes. Tables 3 and 4 report men’s descriptive TDpM quantile benchmarks for the full sample and for the specification excluding bouts shorter than 90 s, respectively. Tables 5 and 6 report the corresponding women’s benchmarks.

**Table 3:** Men’s Descriptive TDpM Quantile Benchmarks, Full Sample (units: takedowns·min-1)

Weight class	*n*	P25	P50	P75	P90
Lightweight	2854	0.000	0.000	0.200	0.333
Welterweight	2742	0.000	0.000	0.158	0.328
Middleweight	2246	0.000	0.000	0.162	0.333
Featherweight	1662	0.000	0.000	0.149	0.333
Bantamweight	1514	0.000	0.000	0.133	0.279
Heavyweight	1502	0.000	0.000	0.120	0.299
Light Heavyweight	1472	0.000	0.000	0.133	0.333
Flyweight	796	0.000	0.067	0.200	0.344

P50, P75, and P90 represent the 50th, 75th, and 90th percentiles of TDpM, respectively. Catchweight bouts were not listed separately because they do not correspond to a fixed weight class

**Table 4:** Men’s descriptive TDpM quantile benchmarks, excluding bouts < 90 s (units: takedowns·min-1)

Weight class	*n*	P25	P50	P75	P90
Lightweight	2674	0.000	0.000	0.200	0.333
Welterweight	2548	0.000	0.000	0.166	0.326
Middleweight	2058	0.000	0.000	0.187	0.333
Featherweight	1556	0.000	0.040	0.169	0.332
Bantamweight	1418	0.000	0.040	0.140	0.289
Light Heavyweight	1330	0.000	0.000	0.133	0.339
Heavyweight	1308	0.000	0.000	0.133	0.300
Flyweight	762	0.000	0.067	0.200	0.333

Excluding bouts shorter than 90 s reduces the influence of extremely short contests on density-based estimates. Catchweight bouts were not listed separately because they do not correspond to a fixed weight class

**Table 5:** Women’s Descriptive TDpM Quantile Benchmarks, Full Sample (units: takedowns·min-1)

Weight class	*n*	P25	P50	P75	P90
Women’s Strawweight	714	0.000	0.067	0.133	0.267
Women’s Flyweight	530	0.000	0.067	0.133	0.267
Women’s Bantamweight	472	0.000	0.020	0.133	0.252
Women’s Featherweight	60	0.000	0.000	0.107	0.277

Given the relatively limited sample size and the smaller number of divisions in the women’s dataset, these values are intended as descriptive quantile benchmarks. Featherweight values are reported for completeness because this division met the predefined n > = 30 inclusion threshold, but upper-percentile estimates should be interpreted cautiously because of the smaller sample size relative to other women’s divisions

**Table 6:** Women’s descriptive TDpM quantile benchmarks, excluding bouts < 90 s (units: takedowns·min-1)

Weight class	*n*	P25	P50	P75	P90
Women’s Strawweight	692	0.000	0.067	0.133	0.267
Women’s Flyweight	520	0.000	0.067	0.133	0.267
Women’s Bantamweight	450	0.000	0.067	0.133	0.251
Women’s Featherweight	56	0.000	0.000	0.126	0.277

These values are descriptive quantile benchmarks and should be interpreted in the context of the shorter observation window and smaller number of women’s divisions. Featherweight values are reported for completeness because this division met the predefined n > = 30 inclusion threshold, but upper-percentile estimates should be interpreted cautiously because of the smaller sample size relative to other women’s divisions. They should not be used to support strong between-division inferences

Across multiple divisions, TDpM showed a zero-inflated distribution, with P25 and/or P50 values equal to zero in several groups. This pattern indicates that many fighter-bout observations included no successful takedowns, while higher percentile values captured the upper tail of takedown-related output. For this reason, P50, P75, and P90 values were reported as descriptive reference points rather than prescriptive thresholds.

In men, the upper percentile values were generally higher in lighter or mid-weight divisions than in the heaviest divisions, consistent with the distributional patterns shown in Fig. 1. In women, P50, P75, and P90 values were relatively similar across the available divisions, consistent with the non-significant between-division tests reported in Table 2.

### Exploratory winner-loser differences in grappling-related performance indicators

**Fig. 5:**
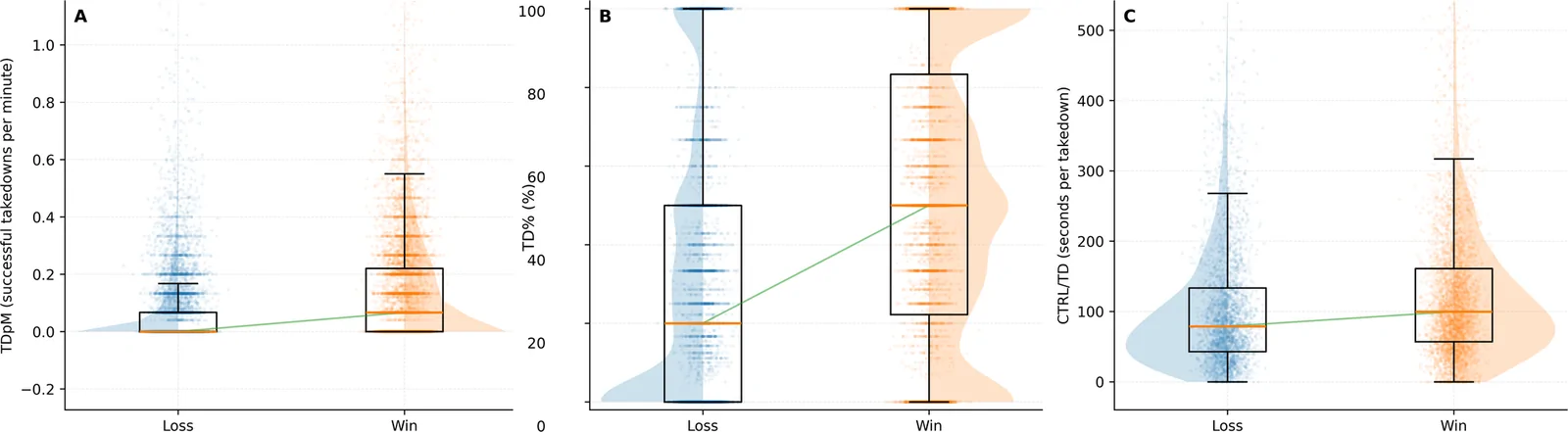
Winner–loser differences in grappling-related performance indicators. Panel A shows TDpM distributions for winners and losers. Panel B shows takedown success rate (TD%) expressed as a percentage. Panel C shows control time per successful takedown (CTRL/TD). Points represent individual fighter–bout observations, boxes indicate medians and interquartile ranges, and shaded density areas show distributional shape. Green lines connect group medians

Winner-loser differences were examined for three grappling-related indicators: TDpM, takedown success rate (TD%), and control time per successful takedown (CTRL/TD). The distributions are shown in Fig. 5, and the corresponding medians and interquartile ranges are summarized in Table 7.

**Table 7:** Winner-loser comparisons in grappling-related performance indicators (median [IQR])

Metric	Winners: Win (median [IQR])	Losers: Loss (median [IQR])	Direction
TDpM (takedowns·min⁻¹)	0.0667 [0.0000, 0.2206]	0.0000 [0.0000, 0.0673]	Win > Loss
TD% (%)	50.0 [22.2, 83.3]	20.0 [0.0, 50.0]	Win > Loss
CTRL/TD (s per takedown)	99.75 [57.00, 161.08]	78.88 [43.00, 133.38]	Win > Loss

TD% is presented as a percentage and was calculated only for bouts with at least one takedown attempt (TDatt > 0). CTRL/TD was calculated only for bouts with at least one successful takedown (TDsucc > 0)

Winners showed higher median TDpM than losers, indicating a higher time-normalized rate of successful takedowns among winning fighters. Winners also showed higher TD% and higher CTRL/TD than losers. These results indicate that the observed winner-loser differences were not limited to successful takedown density alone, but also involved execution efficiency and post-takedown control returns.

These comparisons were exploratory and outcome-related. They should be interpreted as descriptive winner-loser contrasts rather than evidence that TDpM, TD%, or CTRL/TD independently determine bout outcomes.

## Discussion

Using a large historical UFC dataset, this study examined takedown density (TDpM) as a time-normalized takedown performance indicator in professional mixed martial arts [14, 21]. The revised framing is intentionally descriptive: TDpM captures the rate of successful takedowns per unit time and should be interpreted as one grappling-related performance measure rather than a comprehensive measure of total external load [1, 3–5, 8]. Four main findings emerged. First, men’s annual mean TDpM declined across the full historical window, and this direction remained evident in the rule-era sensitivity analysis excluding pre-2002 observations. Second, men’s TDpM differed across weight classes, with heavier divisions generally showing lower values than several lighter and mid-weight divisions, whereas women’s between-division differences were not significant. Third, TDpM distributions were zero-inflated and right-skewed, supporting the use of percentile-based descriptive summaries. Fourth, winners showed higher TDpM, takedown success rate, and control time per successful takedown than losers, indicating outcome-related differences across density, efficiency, and post-takedown control.

### Temporal trends in TDpM

The declining trend in men’s TDpM suggests that successful takedown output per minute has decreased over the long-term development of UFC competition. Importantly, the negative direction remained in the 2002–2025 sensitivity analysis, indicating that the full-period decline was not solely attributable to early pre-unified-rules observations. This strengthens the descriptive evidence that men’s takedown density has changed across competitive eras. Such patterns should be interpreted in the context of evolving UFC tactical strategies and changes in rules or judging criteria [16, 18, 19, 26, 27]. They should also be considered alongside the multidimensional physical demands and pacing characteristics of MMA competition [1, 8, 28].

Several non-exclusive explanations may account for this pattern. As MMA has matured, athletes may have become more selective in their takedown entries because unsuccessful attempts can create counterattacking opportunities, energy costs, and positional risk [1, 2, 15, 16]. Improvements in takedown defense, cage wrestling, distance management, and striking-grappling integration may also reduce the frequency of successful takedowns per minute, even if grappling remains tactically important [10–13]. Therefore, a decline in TDpM should not be interpreted as a reduced importance of takedowns, but rather as a possible shift in the frequency, timing, and efficiency of successful takedown execution.

The women’s temporal results should be interpreted more cautiously. Women’s UFC divisions were introduced later, producing a shorter observation window and smaller annual samples. The non-significant annual trend in women’s TDpM therefore indicates relative stability within the available data rather than definitive evidence that women’s takedown density has not changed.

### Weight-class differences in TDpM

The men’s weight-class analysis showed significant between-division differences in TDpM, although the effect sizes were small. This indicates that weight class contributes to TDpM variability but does not dominate fighter-to-fighter or bout-to-bout variation. The main pattern was not a smooth monotonic gradient across all divisions; rather, heavier divisions, particularly Heavyweight and Light Heavyweight, generally showed lower TDpM than several lighter and mid-weight divisions. Previous MMA time-motion and technical-tactical studies have similarly shown that movement tempo, technical ratios, and bout structure may differ by weight division, sex, and round context [15, 16].

This pattern is consistent with known differences in movement tempo, exchange frequency, and energetic cost across weight classes. Lighter divisions may involve faster transitions, more repeated entries, and more frequent resets, whereas heavier divisions may rely more on lower-frequency but higher-impact exchanges [15, 16]. However, these interpretations should remain cautious because TDpM captures only successful takedowns and does not include attempted takedowns, failed entries, defensive wrestling, or positional context.

Weight-class differences in combat sports should also be considered in the broader context of weight management [29–34]. Rapid weight loss and post-weigh-in weight regain are common across combat sports and grappling disciplines, including sambo and grappling-based events [29, 30], and may influence physiological readiness, pacing, and technical expression [31–33]. Nevertheless, the present dataset did not include individual weight-cutting practices, hydration status, or fight-week body-mass changes. Therefore, weight management should be interpreted as a contextual factor rather than a directly tested mechanism in this study.

In women, TDpM did not differ significantly across available weight classes and effect sizes were close to zero. This may reflect the shorter history of women’s UFC divisions, smaller sample sizes, fewer divisions, and greater influence of individual style or matchup characteristics. Previous technical-tactical analyses of female MMA also suggest that women’s competitive profiles should be interpreted with attention to sample size, division development, and sex-specific tactical contexts [16, 35]. Accordingly, women’s division-level results are best interpreted as descriptive references rather than strong evidence of stable structural differences between weight classes.

### Descriptive quantile benchmarks and distributional interpretation

The descriptive quantile benchmarks provide an interpretable way to summarize TDpM in a distribution that is highly zero-inflated and right-skewed. In several divisions, P25 and/or P50 values were equal to zero, indicating that many fighter-bout observations included no successful takedowns. Under such conditions, means alone may overstate the typical takedown profile because they are sensitive to upper-tail observations. From a performance-analysis perspective, percentile-based summaries can help contextualize sport-specific performance indicators within their distributional structure [14, 21]. Reporting effect sizes alongside statistical tests also supports interpretation beyond p-values alone [23, 36].

These quantile values should be interpreted as descriptive performance-analysis references, not as prescriptive training thresholds. For example, P75 and P90 values may help analysts identify unusually high takedown-density performances within a given division, but they do not specify how a fighter should train or how training load should be prescribed [3–5]. Their value lies in providing a transparent reference distribution against which future competition analyses, scouting reports, or longitudinal descriptive comparisons can be interpreted [14, 21].

The dual reporting of full-sample values and values excluding bouts shorter than 90 s also helps distinguish broad ecological description from a more conservative density estimate. Very short bouts can produce inflated per-minute values, so the sensitivity specification provides a useful comparison when interpreting upper-percentile TDpM values.

### Exploratory winner-loser differences in grappling-related indicators

The winner-loser comparisons showed that winners had higher TDpM, takedown success rate, and control time per successful takedown than losers. This pattern suggests that successful takedown output, execution efficiency, and post-takedown control returns are all relevant to grappling-related performance profiles in MMA [10–13]. These outcome-related differences are consistent with previous MMA studies showing that grappling activity, technical accuracy, phase-specific actions, and outcome-related technical-tactical variables are associated with winning profiles [10–13]. Importantly, the result does not imply that takedown density alone determines bout outcome. Rather, TDpM should be interpreted together with efficiency and control-related variables.

These findings are consistent with the broader view that MMA performance is multidimensional [1, 2, 8, 14]. A fighter may record high takedown density but gain limited competitive advantage if attempts are inefficient, if control time is short, or if the opponent rapidly returns to standing. Conversely, lower-frequency takedown output may still be meaningful when successful takedowns are well timed and lead to sustained control. Therefore, TDpM is most informative when interpreted as part of a broader grappling-related profile rather than as a standalone predictor of victory [10–13].

Because the present analysis was retrospective and observational, the winner-loser comparison should be interpreted as an exploratory outcome-related contrast. It does not establish causality and does not adjust for all potential confounders, such as opponent quality, fighting style, round phase, judging criteria, tactical intention, or match-specific context.

### Practical implications

The practical value of TDpM lies in its simplicity and interpretability as a competition-derived descriptive performance indicator [14, 21]. For analysts and coaches, TDpM can provide a time-normalized summary of successful takedown output that is comparable across bouts of different durations. However, it should not be used in isolation. TDpM should be interpreted together with takedown attempts, success rate, control time, positional advancement, opponent style, and bout context [10–14, 21].

The percentile benchmarks reported in this study may serve as reference values for performance analysis within sex and weight-class groups [14, 21]. They can help contextualize whether a fighter’s takedown output is typical, relatively high, or located in the upper tail of the observed UFC distribution. Such benchmarks may be useful for post-bout analysis, scouting, and descriptive longitudinal tracking. However, because the present study did not analyze training sessions or intervention responses, these values should not be interpreted as empirically tested training-prescription thresholds [3–5].

Future applied use of TDpM would require additional data from training environments, video-coded technical sequences, and repeated within-athlete observations. Such work would be necessary before TDpM could be incorporated into formal training-monitoring systems [3–6, 37, 38]. MMA-specific workload studies and combat-sport sensor research also indicate that monitoring systems require repeated observations, contextual interpretation, and clear links between external outputs, internal responses, and adaptation [7–9, 28].

### Strengths and limitations

This study has several strengths. First, it used a large historical UFC dataset covering 8,461 bouts and 16,922 fighter-bout observations, allowing long-term temporal and division-level patterns to be examined [20]. Second, analyses were stratified by sex and weight class, which is important given the different developmental histories of men’s and women’s UFC divisions and known variation in technical-tactical profiles across divisions [15, 16, 35]. Third, the use of percentile-based summaries is appropriate for TDpM because the distribution is zero-inflated and right-skewed [14, 21]. Fourth, sensitivity analyses excluding bouts shorter than 90 s and excluding pre-2002 men’s observations improved the transparency of temporal interpretation in relation to rule-era concerns [18, 19, 26, 27].

Several limitations should also be acknowledged. First, TDpM captures only one takedown-related dimension of grappling performance and does not represent the full physical or technical demands of MMA competition [1, 8]. Total external load would require broader information on striking, clinching, defensive actions, locomotor activity, and other physical work [3–5]. Second, the study relied on an openly available secondary dataset compiled from publicly accessible UFC Stats records. Although the dataset is released under a CC0 Public Domain license [20], technical actions were not independently re-coded from original fight videos; future studies should incorporate independent video coding and reliability assessment [21, 36, 39, 40]. Third, the dataset did not include detailed contextual variables such as takedown type, cage location, round phase, stance matchup, opponent quality, or tactical intention; such variables are important in technical-tactical and phase-specific MMA performance analysis [11–13, 17, 19, 39]. Fourth, although the 2002–2025 sensitivity analysis addressed part of the rule-era concern, MMA rules, judging criteria, and competitive practices have continued to evolve over time [18, 19, 26, 27]. Finally, winner-loser comparisons were observational and do not establish causality.

### Future directions

Future research should extend this descriptive framework using richer technical-tactical data. Video-coded analyses could distinguish attempted and successful takedowns, entry type, setup actions, cage versus open-space context, round phase, and post-takedown positional advancement [11–13, 39, 41]. Integrating takedown density with striking volume, clinch exchanges, defensive wrestling, and control sequences would provide a more complete picture of grappling-related performance [10–14, 21].

Longitudinal studies using training data are also needed. Repeated within-athlete observations from sparring, technical drills, and competition preparation would allow researchers to examine whether TDpM changes across training cycles and whether such changes relate to performance outcomes [28, 42]. Such studies would be required before TDpM could be used as part of a formal training-monitoring or prescription framework [3–6, 37, 38]. Sensor-based and time-motion approaches could further complement TDpM by capturing grappling-specific workload beyond source-recorded bout statistics [7–9].

Finally, future work could apply era-specific modeling to better account for changes in rules, judging criteria, tactical norms, and athlete preparation. This would help clarify whether the long-term decline in men’s TDpM reflects tactical adaptation, rule-era effects, changing athlete skill profiles, or a combination of these factors [16, 18, 19, 26, 27].

## Conclusions

This retrospective analysis of 8,461 UFC bouts examined TDpM as a time-normalized indicator of takedown-related performance in professional MMA. Men’s TDpM declined across the historical observation window, and this negative direction remained evident after excluding pre-2002 observations. Men also showed significant weight-class differences, with heavier divisions generally displaying lower TDpM than several lighter and mid-weight divisions. In contrast, women’s TDpM showed no significant between-division differences in the available sample. TDpM distributions were zero-inflated and right-skewed, supporting the use of percentile-based descriptive benchmarks. Winners showed higher TDpM, takedown success rate, and control time per successful takedown than losers, but these comparisons should be interpreted as outcome-related contrasts rather than causal evidence. Overall, TDpM provides a simple and interpretable reference measure for takedown-related performance analysis. Future studies incorporating video-coded context and training data are needed to clarify its broader applied value.

## Supplementary Information


Supplementary Material 1


## Data Availability

The datasets analyzed during the current study are publicly available from the official UFC Stats website (https://ufcstats.com/). The processed dataset and analysis code used to generate the results are available from the corresponding author on reasonable request.
