# Supplementary material for: Temporal trends and weight-class differences in takedown density in professional mixed martial arts: a retrospective analysis of 8,461 UFC bouts, 1997–2025
Source: BMC Sports Sci Med Rehabil. 2026 Jun 18;18:385. doi: 10.1186/s13102-026-01804-8 (PMC13523191; doi:10.1186/s13102-026-01804-8)
Supplement: Supplementary file 1 [file 13102_2026_1804_MOESM1_ESM.docx]

**Supplementary Material S1**

**Methods and Statistical Details**

This supplementary file provides additional methodological information on data source, variable operationalisation, data cleaning, sensitivity analyses, and statistical procedures used in the main manuscript. It also reports the Dunn-Holm post hoc pairwise comparisons supporting the weight-class analyses.

# S1.1 Data source and unit of analysis

The study used the publicly available Kaggle dataset “UFC 2025 Dataset: Fights, Fighters & Events”, released under a CC0 Public Domain license. The dataset is an openly released compilation of UFC competition records originally derived from publicly accessible UFC Stats records.

The unit of analysis was the fighter-bout observation. Each retained bout was converted into two fighter-bout observations, one for each competitor. After data cleaning, the analytical dataset included 8,461 UFC bouts and 16,922 fighter-bout observations, comprising 15,146 men’s observations and 1,776 women’s observations. The primary analytical window covered 1997-2025 for men and 2013-2025 for women, corresponding to the available UFC competition history represented in the cleaned dataset for each sex.

# S1.2 Key variables and operational definitions

All variables were computed at the fighter-bout level unless otherwise stated.

1. Takedown density (TDpM): TDpM = TDsucc / Tbout, where TDsucc denotes the number of successful takedowns recorded in the source dataset, and Tbout denotes bout duration in minutes.

2. Takedown success rate (TD%): TD% = (TDsucc / TDatt) × 100, where TDatt denotes the number of attempted takedowns. TD% was calculated only for fighter-bout observations with TDatt > 0.

3. Control time per successful takedown (CTRL/TD): CTRL/TD = control time (seconds) / TDsucc, where control time represents the total control time recorded for a fighter-bout observation. CTRL/TD was calculated only for observations with TDsucc > 0.

4. Bout duration: Bout duration was calculated in minutes using the total recorded fight time, including completed rounds and the time of stoppage where applicable.

5. Sex and weight class: Sex and weight-class information were obtained from the source dataset metadata and harmonized at the fighter-bout level. Analyses were stratified by sex because women’s UFC divisions were introduced later and had a shorter observation window than men’s divisions.

# S1.3 Data cleaning and quality-control rules

The following rules were applied to ensure consistent computation and valid denominators:

(1) Records with missing or invalid bout duration were excluded from analyses requiring time-normalized variables.

(2) Records with missing takedown success counts were excluded from TDpM analyses.

(3) TD% was calculated only for observations with at least one takedown attempt (TDatt > 0).

(4) CTRL/TD was calculated only for observations with at least one successful takedown (TDsucc > 0).

(5) For inferential weight-class comparisons, divisions with at least 30 fighter-bout observations were included. This threshold was used to reduce small-sample instability rather than to imply formal measurement reliability.

(6) Catchweight observations were retained in inferential comparisons when they met the n ≥ 30 criterion, but catchweight was not presented as a fixed division in the descriptive benchmark tables because it does not correspond to a stable UFC weight class.

(7) Technical variables were used as recorded in the source dataset. The study did not independently re-code original fight videos; therefore, takedown-related variables should be interpreted as source-recorded competition statistics rather than independently verified video-coded events.

# S1.4 Data-processing accuracy check

To assess data-processing accuracy from the source CSV files into the analytical dataset, a random 5% subset of retained bouts (n = 423) was re-checked using an independent extraction script and compared against the primary analytical dataset. The checked fields included bout duration, weight class, winner-loser status, successful takedowns, attempted takedowns, and control time. No discrepancies were identified across the checked fields. This procedure verified internal data-processing reproducibility from the same source CSV files; it does not constitute an independent video-coding reliability assessment of the underlying UFC Stats records.

# S1.5 Sensitivity analysis excluding extremely short bouts (<90 s)

Density-based metrics can be inflated in extremely short bouts, such as early stoppages. To evaluate whether the main TDpM patterns were influenced by very short contests, a sensitivity analysis was performed by excluding bouts shorter than 90 seconds. Core analyses, including temporal trend estimation, weight-class comparisons, and percentile-based descriptive summaries, were repeated under this criterion. The purpose of this sensitivity specification was to evaluate robustness to short-bout inflation, not to redefine ecological competition demands.

# S1.6 Rule-era sensitivity analysis excluding pre-2002 men’s observations

Because the early UFC period included rule and format differences relative to the modern unified-rules era, an additional men’s temporal sensitivity analysis was conducted by excluding pre-2002 observations. This analysis was used to evaluate whether the long-term men’s TDpM trend was driven primarily by the early pre-unified-rules period. The 2002-2025 specifications reported in the main manuscript should therefore be interpreted as rule-era sensitivity analyses rather than as evidence that all post-2002 competitions were governed by fully identical rule and judging environments.

# S1.7 Trend analysis based on annual means

For temporal analyses, annual mean TDpM was calculated separately for men and women. Linear regression was then used to estimate the annual change in mean TDpM, with calendar year as the predictor and annual mean TDpM as the outcome. The regression slope, correlation coefficient r, and p value were reported for each temporal specification.

The correlation coefficient r reflects the association between calendar year and annual mean TDpM. It should not be interpreted as a correlation computed at the individual fighter-bout level.

# S1.8 Weight-class differences: Kruskal-Wallis test and effect size

Given the non-normal, zero-inflated, and right-skewed distribution of TDpM, between-division comparisons were conducted using the Kruskal-Wallis test. Epsilon-squared (ε²) was reported as an effect-size estimate to quantify the proportion of rank-based variability associated with weight class. Analyses were performed under both the full-sample specification and the specification excluding bouts shorter than 90 seconds.

# S1.9 Post hoc pairwise comparisons: Dunn test with Holm correction

When the Kruskal-Wallis test indicated a significant omnibus difference, Dunn’s post hoc pairwise comparisons were used to identify pairwise differences across divisions. Holm correction was applied to control the family-wise error rate. Full pairwise comparison results are reported in Tables S1 and S2.

Catchweight met the n ≥ 30 criterion and was therefore included in pairwise inferential comparisons. However, catchweight was not listed in the main descriptive benchmark tables because it is not a fixed UFC weight class.

# S1.10 Percentile-based descriptive TDpM benchmarks

Sex- and division-specific percentile values were calculated to summarize TDpM distributions. P50, P75, and P90 were reported as descriptive quantile benchmarks representing typical, higher, and upper-tail TDpM values within each sex and weight class.

These values should be interpreted as descriptive performance-analysis reference points. They should not be interpreted as prescriptive training thresholds or validated training-monitoring zones.

# S1.11 Software and reproducibility

All analyses and visualizations were performed using Python 3.x. Data ingestion, cleaning, variable computation, non-parametric testing, multiple-comparison adjustment, regression analysis, percentile estimation, and figure generation were conducted using standard Python scientific-computing libraries. Processed analytical data and Python analysis code are available from the corresponding author upon reasonable request.

# Table S1. Dunn-Holm post hoc pairwise comparisons for men’s weight classes, excluding bouts shorter than 90 seconds.

Main pairwise specification: excluding bouts <90 s; divisions with n ≥ 30.

| Weight class 1 | n1 | Weight class 2 | n2 | Z | Holm-adjusted p | Significant (p<0.05) |
| --- | --- | --- | --- | --- | --- | --- |
| Bantamweight | 1418 | Catchweight | 148 | -0.857 | 1.000 | No |
| Bantamweight | 1418 | Featherweight | 1556 | -0.437 | 1.000 | No |
| Bantamweight | 1418 | Flyweight | 762 | -2.743 | 0.134 | No |
| Bantamweight | 1418 | Heavyweight | 1308 | 4.485 | <0.001 | Yes |
| Bantamweight | 1418 | Light Heavyweight | 1330 | 3.293 | 0.026 | Yes |
| Bantamweight | 1418 | Lightweight | 2674 | -0.608 | 1.000 | No |
| Bantamweight | 1418 | Middleweight | 2058 | 0.294 | 1.000 | No |
| Bantamweight | 1418 | Welterweight | 2548 | -0.377 | 1.000 | No |
| Catchweight | 148 | Featherweight | 1556 | 0.674 | 1.000 | No |
| Catchweight | 148 | Flyweight | 762 | -0.548 | 1.000 | No |
| Catchweight | 148 | Heavyweight | 1308 | 2.836 | 0.105 | No |
| Catchweight | 148 | Light Heavyweight | 1330 | 2.305 | 0.381 | No |
| Catchweight | 148 | Lightweight | 2674 | 0.64 | 1.000 | No |
| Catchweight | 148 | Middleweight | 2058 | 0.989 | 1.000 | No |
| Catchweight | 148 | Welterweight | 2548 | 0.727 | 1.000 | No |
| Featherweight | 1556 | Flyweight | 762 | -2.423 | 0.292 | No |
| Featherweight | 1556 | Heavyweight | 1308 | 5.011 | <0.001 | Yes |
| Featherweight | 1556 | Light Heavyweight | 1330 | 3.796 | 0.004 | Yes |
| Featherweight | 1556 | Lightweight | 2674 | -0.123 | 1.000 | No |
| Featherweight | 1556 | Middleweight | 2058 | 0.78 | 1.000 | No |
| Featherweight | 1556 | Welterweight | 2548 | 0.11 | 1.000 | No |
| Flyweight | 762 | Heavyweight | 1308 | 6.476 | <0.001 | Yes |
| Flyweight | 762 | Light Heavyweight | 1330 | 5.478 | <0.001 | Yes |
| Flyweight | 762 | Lightweight | 2674 | 2.514 | 0.239 | No |
| Flyweight | 762 | Middleweight | 2058 | 3.145 | 0.040 | Yes |
| Flyweight | 762 | Welterweight | 2548 | 2.681 | 0.154 | No |
| Heavyweight | 1308 | Light Heavyweight | 1330 | -1.187 | 1.000 | No |
| Heavyweight | 1308 | Lightweight | 2674 | -5.687 | <0.001 | Yes |
| Heavyweight | 1308 | Middleweight | 2058 | -4.575 | <0.001 | Yes |
| Heavyweight | 1308 | Welterweight | 2548 | -5.422 | <0.001 | Yes |
| Light Heavyweight | 1330 | Lightweight | 2674 | -4.342 | <0.001 | Yes |
| Light Heavyweight | 1330 | Middleweight | 2058 | -3.284 | 0.026 | Yes |
| Light Heavyweight | 1330 | Welterweight | 2548 | -4.086 | 0.001 | Yes |
| Lightweight | 2674 | Middleweight | 2058 | 1.027 | 1.000 | No |
| Lightweight | 2674 | Welterweight | 2548 | 0.27 | 1.000 | No |
| Middleweight | 2058 | Welterweight | 2548 | -0.764 | 1.000 | No |

Note: Pairwise comparisons were conducted using Dunn’s test following a significant Kruskal-Wallis test. Holm correction was applied for multiple comparisons. This table corresponds to the primary pairwise specification excluding bouts shorter than 90 seconds and including divisions with at least 30 fighter-bout observations. Catchweight met the n ≥ 30 criterion and was included in inferential pairwise comparisons, but it was not listed in the main descriptive benchmark tables because it is not a fixed UFC weight class.

# Table S2. Dunn-Holm post hoc pairwise comparisons for men’s weight classes, full-sample specification.

Full-sample pairwise specification.

| Weight class 1 | n1 | Weight class 2 | n2 | Z | Holm-adjusted p | Significant (p<0.05) |
| --- | --- | --- | --- | --- | --- | --- |
| Bantamweight | 1514 | Catchweight | 152 | -1.377 | 1.000 | No |
| Bantamweight | 1514 | Featherweight | 1662 | -0.745 | 1.000 | No |
| Bantamweight | 1514 | Flyweight | 796 | -3.463 | 0.012 | Yes |
| Bantamweight | 1514 | Heavyweight | 1502 | 5.181 | <0.001 | Yes |
| Bantamweight | 1514 | Light Heavyweight | 1472 | 3.72 | 0.005 | Yes |
| Bantamweight | 1514 | Lightweight | 2854 | -1.185 | 1.000 | No |
| Bantamweight | 1514 | Middleweight | 2246 | 0.372 | 1.000 | No |
| Bantamweight | 1514 | Welterweight | 2742 | -0.595 | 1.000 | No |
| Catchweight | 152 | Featherweight | 1662 | 1.07 | 1.000 | No |
| Catchweight | 152 | Flyweight | 796 | -0.389 | 1.000 | No |
| Catchweight | 152 | Heavyweight | 1502 | 3.593 | 0.008 | Yes |
| Catchweight | 152 | Light Heavyweight | 1472 | 2.973 | 0.059 | No |
| Catchweight | 152 | Lightweight | 2854 | 0.954 | 1.000 | No |
| Catchweight | 152 | Middleweight | 2246 | 1.545 | 1.000 | No |
| Catchweight | 152 | Welterweight | 2742 | 1.177 | 1.000 | No |
| Featherweight | 1662 | Flyweight | 796 | -2.903 | 0.070 | No |
| Featherweight | 1662 | Heavyweight | 1502 | 6.043 | <0.001 | Yes |
| Featherweight | 1662 | Light Heavyweight | 1472 | 4.544 | <0.001 | Yes |
| Featherweight | 1662 | Lightweight | 2854 | -0.364 | 1.000 | No |
| Featherweight | 1662 | Middleweight | 2246 | 1.2 | 1.000 | No |
| Featherweight | 1662 | Welterweight | 2742 | 0.239 | 1.000 | No |
| Flyweight | 796 | Heavyweight | 1502 | 7.761 | <0.001 | Yes |
| Flyweight | 796 | Light Heavyweight | 1472 | 6.541 | <0.001 | Yes |
| Flyweight | 796 | Lightweight | 2854 | 2.842 | 0.081 | No |
| Flyweight | 796 | Middleweight | 2246 | 3.975 | 0.002 | Yes |
| Flyweight | 796 | Welterweight | 2742 | 3.293 | 0.021 | Yes |
| Heavyweight | 1502 | Light Heavyweight | 1472 | -1.431 | 1.000 | No |
| Heavyweight | 1502 | Lightweight | 2854 | -7.101 | <0.001 | Yes |
| Heavyweight | 1502 | Middleweight | 2246 | -5.289 | <0.001 | Yes |
| Heavyweight | 1502 | Welterweight | 2742 | -6.47 | <0.001 | Yes |
| Light Heavyweight | 1472 | Lightweight | 2854 | -5.418 | <0.001 | Yes |
| Light Heavyweight | 1472 | Middleweight | 2246 | -3.692 | 0.005 | Yes |
| Light Heavyweight | 1472 | Welterweight | 2742 | -4.803 | <0.001 | Yes |
| Lightweight | 2854 | Middleweight | 2246 | 1.775 | 1.000 | No |
| Lightweight | 2854 | Welterweight | 2742 | 0.697 | 1.000 | No |
| Middleweight | 2246 | Welterweight | 2742 | -1.104 | 1.000 | No |

Note: Pairwise comparisons were conducted using Dunn’s test following a significant Kruskal-Wallis test. Holm correction was applied for multiple comparisons. This table reports the full-sample pairwise specification. Catchweight met the n ≥ 30 criterion and was included in inferential pairwise comparisons, but it was not listed in the main descriptive benchmark tables because it is not a fixed UFC weight class.
